# Spatiotemporal analysis of seasonal trends in land surface temperature within the distribution range of *Moringa peregrina* (Forssk.) in Southern and Southeastern Iran

**DOI:** 10.1371/journal.pone.0306642

**Published:** 2024-07-25

**Authors:** Hossein Piri Sahragard, Peyman Karami

**Affiliations:** 1 Department of Rangeland and Watershed Management, Faculty of Soil and Water, University of Zabol, Zabol, Iran; 2 Department of Environmental Sciences, Faculty of Natural Resources and Environment Sciences, Malayer University, Malayer, Iran; Hanoi University of Mining and Geology, VIET NAM

## Abstract

Temperature fluctuations and related factors are among the main causes of climate change. Understanding the temporal and spatial variations in temperature can shed light on how species respond to climate change. Plants generally persist in suitable microclimates in response to environmental change; however, examining long-term temperature variations within a species’ range can be challenging using field observations. Thermal remote sensing, on the other hand, provides multi-scale time-series data with good coverage and regularity to overcome the challenges associated with field observations in environmental monitoring. Although changes in land surface temperature (LST) affect climate, hydrological processes, land-atmosphere interactions, and ecological activities, this metric has not received much research attention. This study aimed to analyze changes in habitat suitability and microclimatic conditions for *Moringa peregrina*. Seasonal changes in LST within the distribution range of the species were also investigated. To this aim, mean seasonal LST was computed in Google Earth Engine using the daily MODIS/006/MYD13A2 product from 2003 to 2023. Subsequently, a binary habitat suitability map was created based on the true skill statistic (TSS). The Mann-Kendall test was used to analyze seasonal LST trends. Major trends in LST were quantified based on the z-score, and compatibility with habitat suitability was evaluated via GAP analysis and the Kappa index. Seasonal temperature trends were evaluated by comparing each season with the following season using binary comparison. Landforms at presence points were regarded as microclimates and the sensitivity of microclimates to LST was evaluated using two methods: Principal component analysis (PCA) was used to quantify seasonal LST heterogeneity and the random forest (RF) approach was used to evaluate the effect of environmental parameters on habitat suitability within microclimates. The Kappa index revealed a weak overlapping between suitable / unsuitable habitat and the surfaces affected by the trend of changes. Moreover, the suitable habitat of Moringa peregrina in spring, autumn and winter is spatially overlapped by areas that have shown an increasing LST trend, and the presence points have not experienced an increasing temperature trend only in the summer. The findings show that the analysis of seasonal trends in LST provides insights into the effect of LST on habitat suitability and the condition of vegetation. The current study clearly shows that seasonal changes have had a significant impact on the distribution and habitat suitability of *M*. *peregrina*, particularly during summer and winter. Improved habitat suitability and range expansion were observed throughout the year. The study also highlights the role of landforms in regulating temperature. Landforms such as local ridges with minimal temperature fluctuations and regions near the Oman Sea were identified as potential future habitats due to favorable humidity conditions.

## Introduction

Climate changes and global warming are among the main drivers of species distribution shifts at the local and global scales. Mean global temperature increased by about 0.85°C in the 20^th^ century and the impact of climate changes on global biodiversity is expected to intensify in the near future [1]. Some species are unable to adapt to the rapid pace of climate change [2], which necessitates research into the effects of climate change on species distributions [3].

Climate is largely determined by changes and patterns in temperature and precipitation. Consequently, these two factors can also be used to forecast the effects of climate change. Plants are heavily affected by changes in temperature [4], including their growth, development, distribution range, and community composition [5]. Every plant species has a range of ideal temperatures, with higher temperatures within the ideal range leading to increased growth, including longer, thicker shoots and larger leaves [6]. Given the cyclical nature of temperature trends, time scaling can be used to examine the effects of temperature on plants. However, time scaling is often neglected in conservation and ecology. Although time scaling is most commonly applied on a year-to-year basis, shorter time spans, such as months and seasons, can also be utilized [7]. The components of climate change (such as soil moisture, snow cover, evaporation rates, river flows, and lake levels) can be impacted by seasonal variations in temperature and rainfall, which in turn affect the needs of ecological communities [8].

The study of ecological niches can also be conducted over different timeframes to understand how ecological niches are influenced by transient environmental factors such as temperature [9], giving rise to the concept of the temporal ecological niche. Seasons are an ideal timeframe for investigating ecosystems because they bring about significant changes in environmental conditions, impacting ecological processes and dynamics. In addition to affecting abiotic conditions, seasonality can affect species interactions, and community composition and structure [7], along with species-specific variables such as occurrence, developmental cycle, abundance, and environmental interactions [10]. Research has demonstrated that seasonal temperature variations impact the physiology of marine organisms in a variety of ways, including accelerated development and growth, suppressed reproduction, elevated disease prevalence, and elevated mortality [11]. Vein density and photosynthetic capability in certain plants (e.g., *Arabidopsis thaliana*) are associated with possible responses to summer and winter variations [12]. Moreover, seasonal variations in temperature can have an impact on metabolic rates [13]. However, reliable conclusions regarding the effects of temperature require long-term data. Under the influence of climate change, heat waves and other extreme temperature events are projected to occur more frequently and last for longer durations [14]. Additionally, some studies suggest that warm seasons will last longer [15]. Analyzing time-series data of dynamic ecosystem variables over multiple seasons and years will enable researchers to study the behavior of environmental phenomena and evaluate their potential impacts on plant species [16].

One crucial aspect of climate change research is investigating long-term trends in climatic variables [17]. However, many studies overlook the importance of seasonality in ecological processes, primarily due to two reasons: 1-Empirical challenges: Collecting year-round data over multiple years requires substantial time and resource investments, which can be demanding, and 2-Modeling complexity: Analyzing seasonal patterns and trends requires sophisticated mathematical models, which can pose challenges for researchers with limited resources or expertise [18]. Nonetheless, remote sensing (RS) has offered effective solutions to the empirical challenges. Ecologists can now track and examine seasonal variations using RS data and take advantage of their continuous, long-term observations of a variety of environmental factors. Furthermore, modeling complexity has been addressed by advances in modeling approaches and data processing for remote sensing. These changes have culminated in an improved knowledge base regarding the temporal dynamics of ecosystems and their response to environmental change.

One of the important RS products that has not received much attention from researchers is land surface temperature (LST). LST is considered a fundamental indicator of climate and biological processes, as well as a representative measure of weather changes [19]. In addition, LST can be used to understand energy balance, regional climate change, ground and surface water, vegetation phenology, human health, land use, vegetation [20] and atmospheric parameters in areas with sparse data, such as mountainous regions [21]. High LST affects soil surface ecological processes, alters atmosphere-land interactions, reduces plant growth and development, and contributes to heat waves and fires [22]. For example, germination is affected by seasonal fluctuations in LST, especially for nondormant seeds [23]. Additionally, LST influences various plant growth stages, including the transition of seeds from dormancy to active growth, which may be triggered by temperature changes. Therefore, changes in LST are important for temperature-sensitive species [24]. Temperature changes can be a trigger for cambial activity, including the seasonal cycles of activity and dormancy for trees in temperate and cold cliamtes [25]. The difference between LST and atmospheric temperature is often negligible, with a typical variation of ±1°K [26]. Consequently, LST serves as a reliable indicator of the relationship between land surface and atmospheric parameters [27]. However, when using LST as an index to evaluate climate change, it is important to consider the influence of environmental factors, particularly land use patterns.

Prior to investigating the effects of temperature on the distribution of a species, it is necessary to estimate the current range of the species, as changes in range size offer an accurate estimation of the influence of temperature [28]. Species distribution models (SDMs) offer a valuable tool for analyzing and assessing the effects of environmental changes, including temperature, on plant and animal species. These models leverage presence/absence data and establish relationships with environmental variables to predict distribution ranges.

In recent years, the use of modeling tools and SDMs has garnered increasing attention as a fundamental approach to study the impact of climate change on species at various spatial scales [29, 30]. The data used in these scenarios are accessible through global databases such as WorldClim and CHELSA. While CHELSA provides downscaled data, WorldClim relies on interpolation methods [31], leading to potential differences in the data used and subsequent findings [32]. The accuracy of information from these databases is more likely to be compromised in high-altitude areas due to topographic variations [33]. Additionally, the effectiveness of interpolation methods for these areas remains a challenge [34]. Theoretically, RS can address these challenges and offer a variety of data for analyses at different spatial and temporal scales. Regarding the study of LST and its impacts, MODIS satellite imagery offers various data products with high spatial and temporal resolution [35]. For instance, RS data can be used to identify hot spots with maximum LST values, or to analyze estimates from complex climate models [36]. Given the influence of seasonal LST trends on vegetation, green biomass, and the overall quality and quantity of vegetation cover across vast areas, it is crucial to investigate the seasonal and cyclical changes in this index and uncover relevant patterns [37].

The resilience of plant communities is influenced by the timing of germination and the success of recruiting seedlings in response to climatic variations [38]. Plants modify the timing of germination in response to environmental factors to maximize resource acquisition and minimize abiotic stresses. Refuges provide favorable conditions that enable plants to achieve this. A refuge is a location that shelters an organism from temporary stressors in its environment [39, 40]. According to some definitions, refuges play a crucial role in protecting endangered species from harm [41]. These refugial areas may provide essential moisture for certain plant species. Microclimatic refugia, which create locally protected environments from broader climatic conditions, are often utilized by plants with small and isolated populations [42].

Given that plant species have limited mobility compared to animals, they are particularly affected by seasonal variations. Moreover, plant movements are more affected by land surface characteristics, with isolated habitats exhibiting different functions over time. Elevation and its spatial gradient are considered crucial factors in the formation of microclimatic refuges. Variations in elevation create topographic structures that provide suitable microhabitats and shelter for species based on their range of movement. One significant feature generated by elevation gradients and associated factors is landform. Landforms play a vital role in facilitating plant establishment and germination by offering favorable microclimatic conditions.

Landforms, such as plains and mountains at large scales, and valleys and hills at smaller scales [43], are crucial environmental features that influence ecosystem development and material and energy flows [44]. These topographic structures, by affecting key parameters such as elevation and humidity, can impact habitat suitability and dispersal for species with limited mobility [45]. In other words, spatial heterogeneity in environmental conditions, resulting from topographic features and climatic factors, plays a significant role in constraining the distribution ranges of species [46]. However, the distribution of plant and animal species across different landforms, and its relationship with changes in LST and habitat suitability have received less attention [47]. While climate change data is frequently employed in studies on refuges, such data may not be entirely appropriate for assessing refugial areas. Investigating the habitat suitability of *Neurergus derjugini* across different seasons through a combined SDM-LST approach revealed that habitat suitability is influenced by LST. The study indicated that northern populations will be more vulnerable as temperatures rise [47]. Furthermore, an analysis of seasonal LST changes in the habitats of *Salamandra infraimmaculata* showed that LST experienced significant variations in the eastern habitats, And the temperature in the habitat of this species in Iran is higher than other habitats [48].

The genus *Moringa*, belonging to the Moringaceae family, is native to tropical and subtropical regions such as India, northeastern Africa, and southwestern Asia [49]. In Iran, *M*. *peregrina* has been introduced as a drought-resistant plant, but it is now endangered due to climate change, heavy livestock grazing, eradication, over-exploitation, and mismanagement [45]. Despite the significant daily and seasonal variations in soil temperature and their impacts on physical processes such as soil changes and mineralization [50], as well as the influence of vegetation diversity, density, and microclimate changes [51], few studies have utilized LST to monitor the habitat status of this species. However, the combination of SDMs with remote sensing data such as LST and landforms allows for a more accurate evaluation of the impact of environmental variables on habitat suitability for the species. Moreover, identifying the spatial distribution of directional changes in environmental parameters such as LST can contribute to microclimate monitoring and management. Therefore, the current study aims to 1- investigate temperature trends and seasonal LST cycles in *M*. *peregrina* habitats, 2- analyze the role of landform classes in creating microclimates, and 3- study the relationship between habitat suitability and LST changes in the habitats of *M*. *peregrina* in southern and southeastern Iran.

## Materials and methods

### Study area

**Fig 1:**
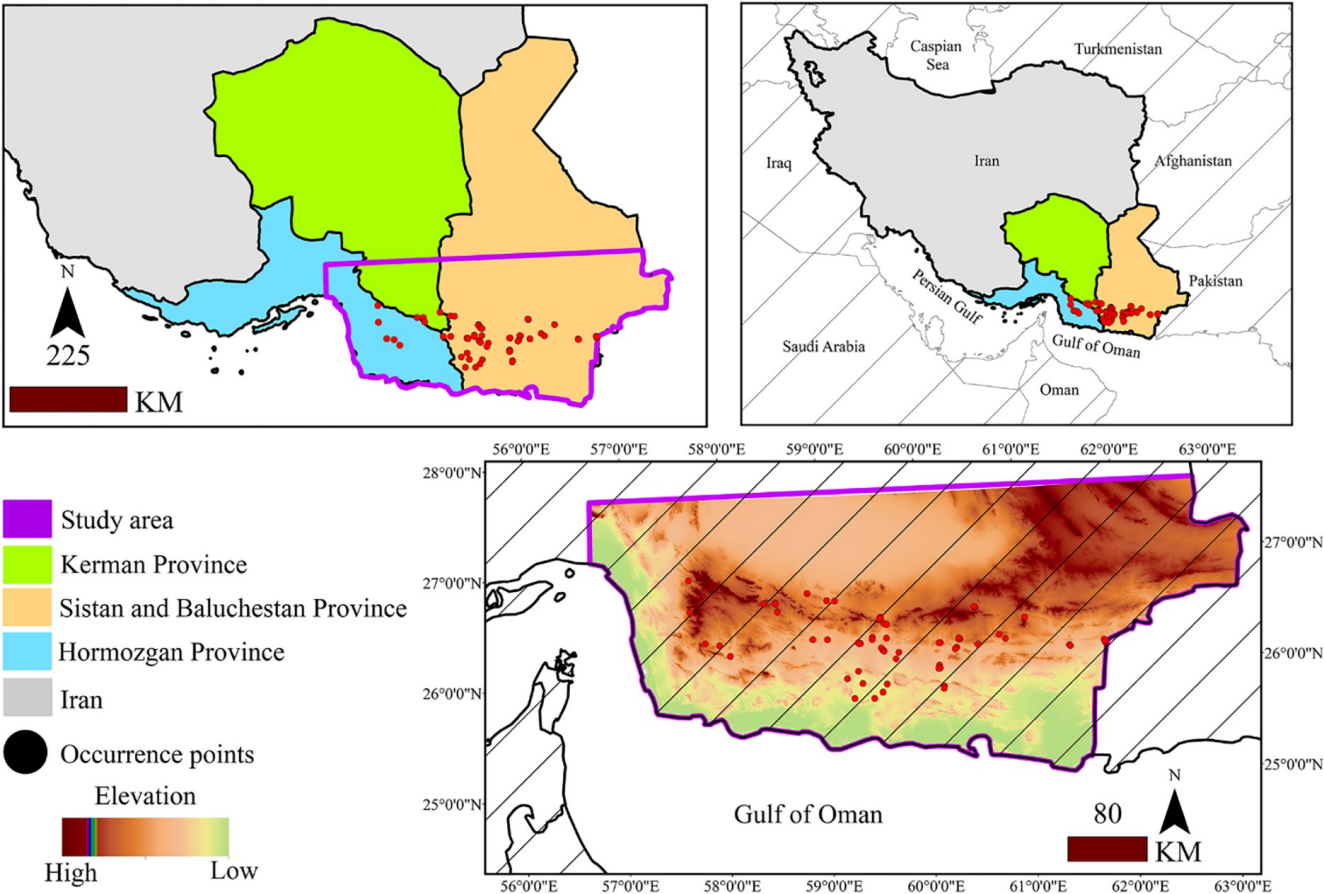
Geographical location of the study area and presence points of *M*. *peregrina*.

The study area covers 137244.90 km^2^ in southeastern iran, including parts of Hormozgan, Sistan and Balochestan, and Kerman provinces (Fig 1). It is bordered by the Oman Sea from the south and the Persian Gulf from the east. The highest points are located in the central, eastern, and southeastern regions and a mountain range is located in the center of the study area. Mean annual precipitation in the study area is 150–200 mm [34]. In order to collect species presence points, distribution limits were extracted from Flora of Iran [52] and Flora Iranica [53]. Afterwards, presence points of *M*. *peregrina* were recorded through field observations using a GPS device. In total, 61 presence points were recorded (Fig 1).

### Habitat suitability modeling

Ensemble habitat suitability modeling for *M*. *peregrina* was conducted according to the literature [46]. Modeling was conducted using 61 presence points and 120 pseudo-absence points. The ensemble model was created through combining the outputs of several models, including classification and regression tree (CART), random forest (RF), generalized paths seeker (GPS), multiple additive regression (mars), and logistic regression (LR). Since a binary map is required to study the impact of temperature changes on habitat suitability [46], a threshold value of the true skill statistic (TSS) was used to convert the habitat suitability map into a binary map. The threshold was computed using presence and pseudo-absence points in SPSS v16. Model performance was evaluated using sensitivity, specificity, classification, and misclassification. Sensitivity represents the percentage of presence points correctly recognized as presence points, and feature represents the number of pseudo-absence points correctly recognized as pseudo-absence points. The ideal model has high sensitivity, specificity, and classification scores and a low misclassification score [54].

### LST and analysis of trends

This study utilized daily data to better examine the influence of temperature changes since temporal thermal variation is thought to be an extrinsic ecological component that affects the variance of species range sizes [55]. Multiple products based on Aqua and Terra satellites offer LST through MODIS products. In this study, LST time series was evaluated using the MODIS-MYD11A1 product. The MYD11A1 product is generated from the level-2 LST product MYD11−L2, in which LST is derived using the generalized split-window LST algorithm [56]. Mean LST was estimated for each season during 2003–2023 and entered into Google Earth Engine [57]. The seasonal scheme in this study pertains to the three-month period, while the seasonal changes correspond to the long-term trend of LST during each season. Next, all maps were entered into TerrSet v2020 for each season as time series and saved in the TSF format in TreeSET. Pre-whitening was not performed since the data was in raster format [48]. Finally, the images were entered into MK analysis. The analysis was performed for four seasons. To assess the significance of changes, the z-core (ZMK) was applied. ZMK has a mean of 0 and a variance of 1 and follows the standard normal distribution. The fluctuations in this statistic indicate the significance of changes in the desired variable [58]. Significance was assessed at the 1% and 5% level using two-tailed tests, corresponding to ZMK scores of ±1.96 and ±2.576, respectively [58]. The thresholds were applied using the raster calculator function in ArcGIS 10.4.1 to determine regions with decreasing or increasing temperature trends and to generate a binary map.

### Analysis of habitat affectability

GAP analysis was conducted to study distribution under the impact of temperature trends. To do this, the binary map of habitat suitability and regions with increasing and decreasing trends were superimposed and the agreement rate for the two maps was evaluated using the Kappa coefficient. The analysis was performed in TreeSET along with CROSSTAB analysis. The Kappa coefficient is based on the overall accuracy of the model’s predictions with respect to the expected accuracy in the random mode. The Kappa coefficient ranges from +1 to -1, where +1 indicates a complete match and zero and smaller values indicate that the model did not perform better than the random mode [59]. In order to study the seasonal cycle of changes in ZMK from spring to summer, summer to fall, fall to winter, and winter to spring, pixel-base comparison was conducted. In this method, ZMK maps were compared pixel-wise using Eq (1) in ArcGIS.


abs(b–a)/max(abs(b‐a)) Eq (1)

Where a and b refer to the ZMK value of the first and second season, respectively. Using this approach, seasonal thermal changes in the landscape were computed. High and low values correspond to the regions with more or less ZMK changes between the two seasons, respectively.

### Analysis of fluctuations in presence areas

We investigated the physical characteristics of land cover that contribute to establishing microclimatic refuges in the context of LST fluctuations and their effects. Microclimatic refuges can be effectively created by various landform classes [60]. Thus, landforms in presence areas were regarded as microclimatic refuges in this study. LST in the locations where the species is present was examined using two methods. The first method examined LST heterogeneity fluctuations across landforms, and the second method examined LST trends in relation to habitat suitability. To investigate LST fluctuations in *M*. *peregrina* habitats, landforms were classified based on a digital elevation model (DEM) and the topographical position index in QGIS 3.16 [61]. Landform classes were calculated using a smaller neighborhood (5×5 cells) and a larger neighborhood (11×11 cells) [45]. Landforms were classified into 10 classes, including: 1 = canyons, 2 = midslope drainages, 3 = upland drainages, 4 = u-shaped valleys, 5 = plains, 6 = open slopes, 7 = upper slopes, 8 = local ridges, 9 = midslope ridges, and 10 = mountain tops. Afterwards, using the extract value to point tool in ArcMap, each presence point was assigned to a landform. In the next step, LST values for all seasons in all years were entered into PCA and LST heterogeneity was estimated for each season based on the top 3 components. Finally, severity of temperature fluctuations was examined for different landforms according to the calculated heterogeneity using the ggplot package. To identify the presence points that have experienced the increasing trend of LST, the extract to point tool was used to extract the ZMK [62]. In the final stage, the relationship between habitat suitability and seasonal trends was analyzed using RF regression. This method has demonstrated strong performance in species distribution studies [63]. In this model, the ZMK values derived for seasonal trends served as the independent variables for each season and habitat suitability at presence points served as the dependent variable. Using this method allowed us to examine the impact of seasonal variations in temperature on the distribution of *M*. *peregrina*. Given the number of presence points (61 points), the final RF model was evaluated using the out-of-bag error statistic [62], R^2^ (RSQ), median absolute deviation (MAD), mean absolute percentage error (MAPE), root-mean-square deviation (RMSE), and mean squared error (MSE) in R 4.1.3.

## Results

### Habitat suitability

**Fig 2:**
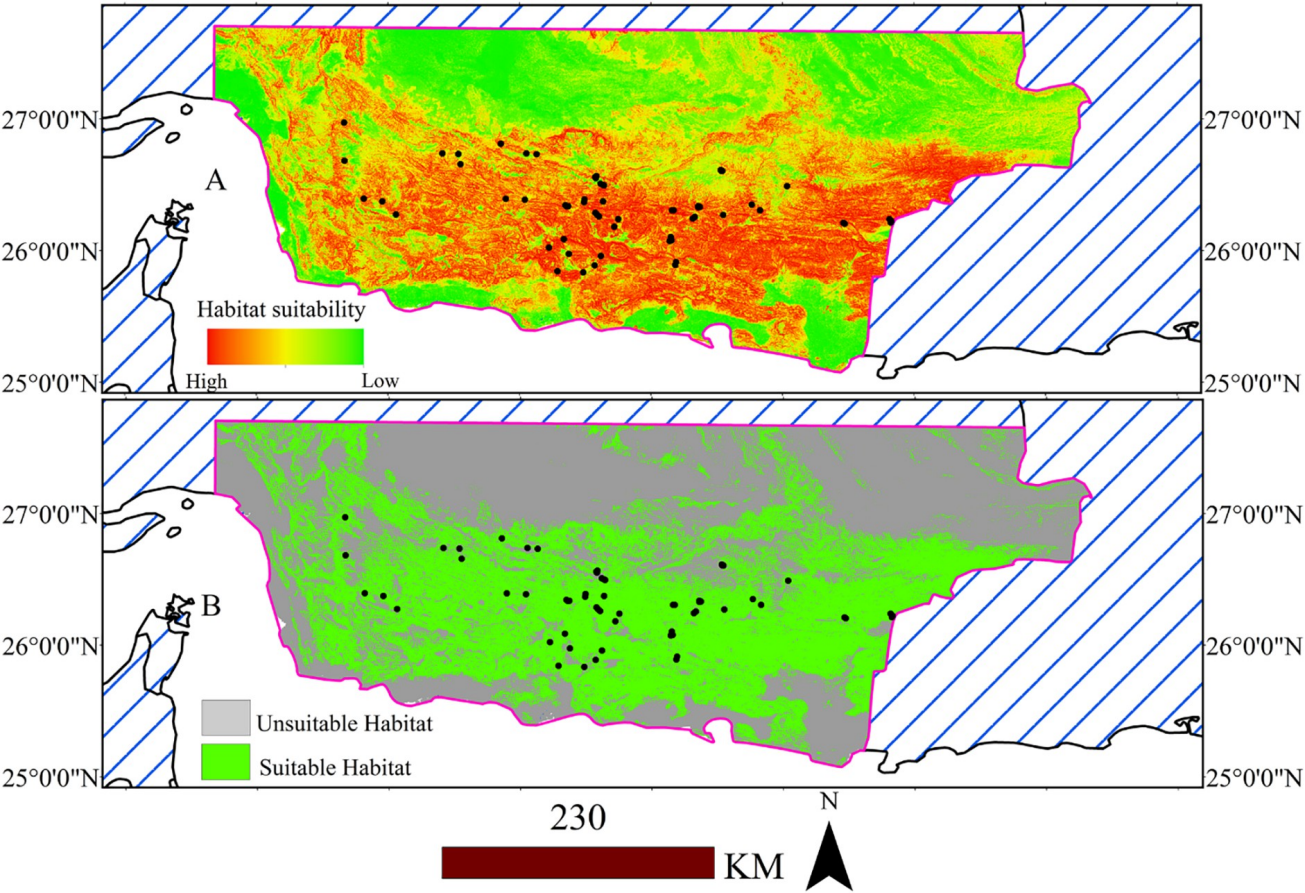
Habitat suitability map (A), and binary habitat suitability map (B) for *M*. *peregrina* in southern and southeastern Iran.

The binary suitability map was generated using TSS>0.487 as the threshold (Fig 2A). Results indicated acceptable performance (sensitivity = 0.984 and specificity = 0.984) (Table 1), showing that more than 98% of the presence and pseudo-absence points were correctly identified as such.

**Table 1:** Model performance for habitat suitability of *M*. *peregrina*.

Statistic	Value	Lower bound (95%)	Upper bound (95%)
**Correct classification**	0.984	0.965	1.000
**Misclassification**	0.016	0.000	0.035
**Sensitivity**	0.984	0.903	1.000
**Specificity**	0.984	0.938	0.999

The presence and absence map demonstrates that the east-facing slopes and the coast of the Oman Sea are more suitable for the species (Fig 2B).

### Seasonal LST cycle

**Fig 3:**
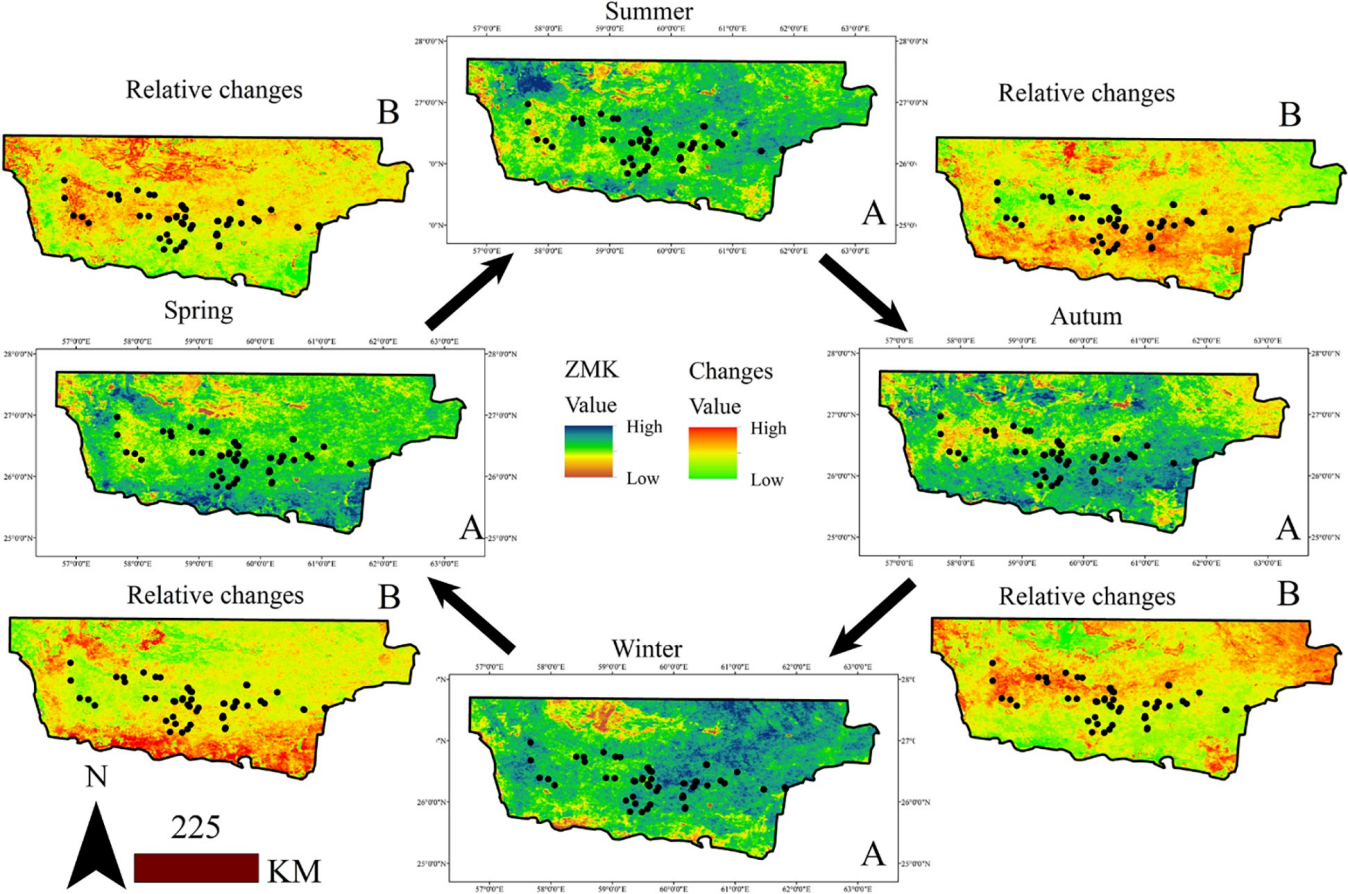
Cyclic changes of LST based on ZMK for each season in *M*. *peregrina* habitats (A) and relative comparison of ZMK changes is shown for each both seasons (B).

Fig 3 presents the ZMK index values, where high and low numerical values indicate increasing and decreasing LST trends, respectively, for each season. In spring, the southern regions and areas adjacent to the Oman Sea experienced the most significant LST increases based on the ZMK index. In contrast, during summer, the increasing LST trends occurred in the northern part of the study area. Furthermore, the southern parts and some northern regions exhibited increasing temperature trends in fall, while the central regions showed a decreasing trend. The largest ZMK index values at the presence points were observed in winter, indicating that most habitats were impacted by increasing temperature trends during winter. The study of the seasonal LST cycle from spring to summer reveals that there is a significant temperature differential in the northern habitats of the species, whereas the lower latitude habitats experience less seasonal temperature variation. During the transition from fall to winter, a larger temperature differential was observed at lower latitudes. The pattern of variations from spring to summer resembles that from fall to winter, with the presence sites in higher latitudes experiencing the largest temperature swings. The transition from summer to autumn follows a similar pattern to that of winter to spring.

### Seasonal LST trends in habitats

**Fig 4:**
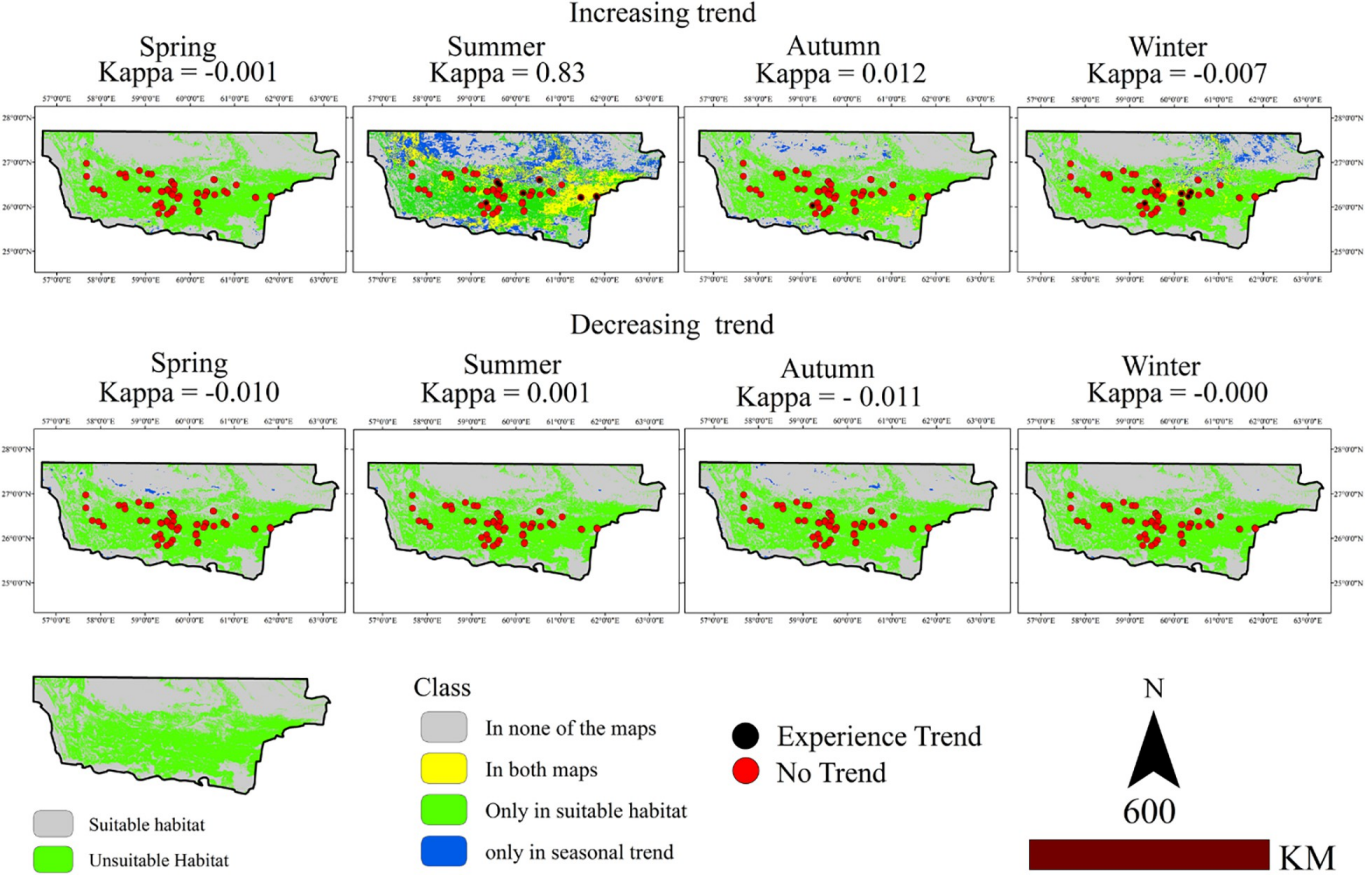
Increasing and decreasing temperature trends in *M*. *peregrina* habitats at 95% confidence level.

Fig 4 presents the superimposed maps of species habitat suitability and regions with increasing and decreasing LST trends, with gray representing regions with no overlap between suitability and trend maps, yellow indicating regions present in both maps, green representing regions existing only in the habitat suitability map, and blue representing regions existing only in the trend maps. The agreement between the suitability and trend maps is displayed by the Kappa coefficient (Fig 4). Black points indicate regions experiencing the trends. Some regions exhibited increasing temperature trends in summer, fall, and winter, whereas no increasing trends were observed in at presence points during spring. No presence points experienced decreasing temperature trends. The Kappa index (0.83) demonstrates a significant overlap between areas with an increasing trend and suitable habitats.

### Seasonal fluctuations of LST index in landforms

**Fig 5:**
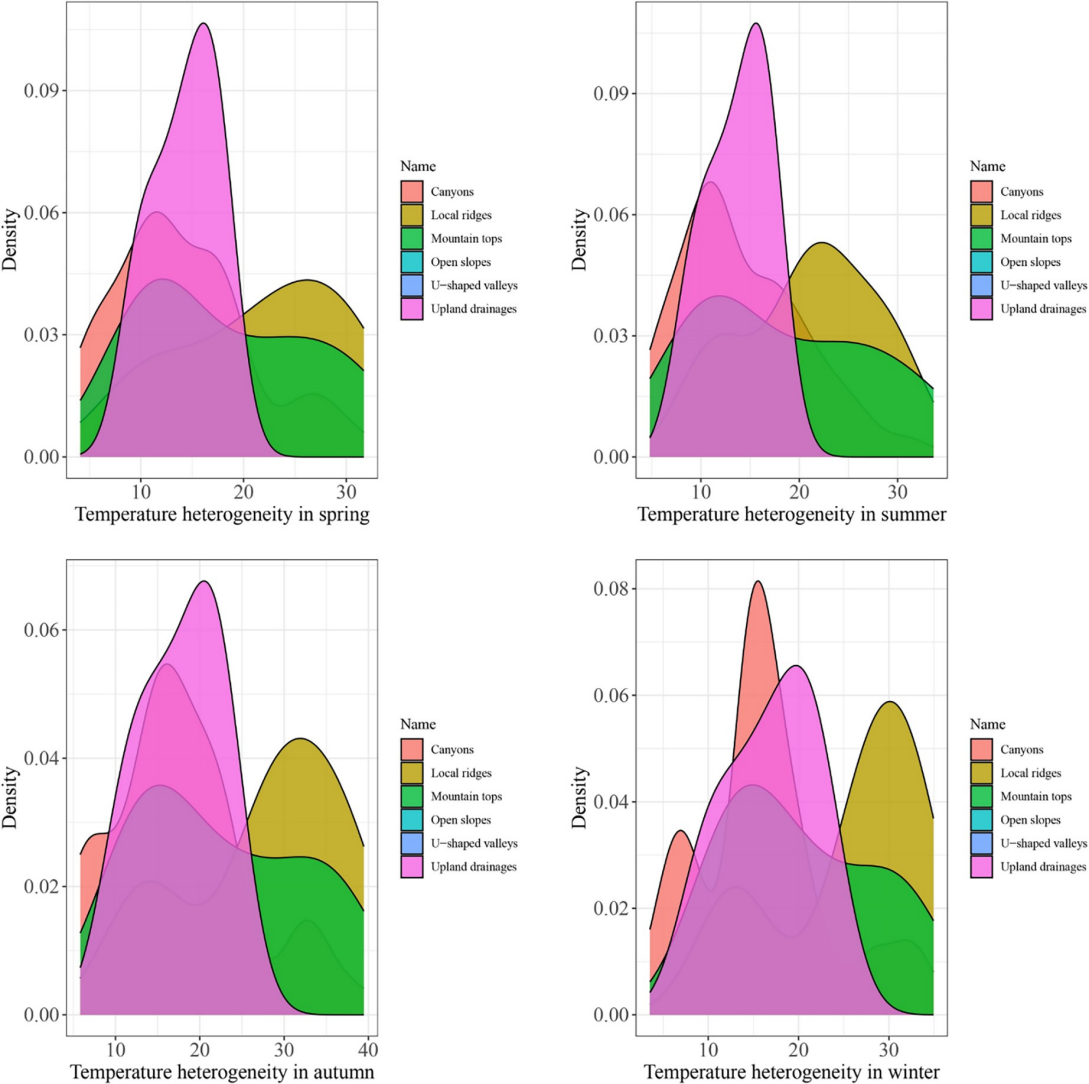
LST heterogeneity in presence areas of *M*. *peregrina* separated by landform class.

LST heterogeneity in the presence areas is shown in Fig 5 using different colors for landform classes. These graphs demonstrate the variation in LST in the species’ presence areas between 2003 and 2023.The x and y axes indicate landform heterogeneity and density, respectively. Upland drainages had the least temperature fluctuations in spring and summer (0–20). In all seasons, mountain tops had a 2-peak response with LST fluctuations of up to 30%, while local ridges experienced small changes in fall and winter. In canyons, LST variations were minimal throughout the year. Spring, summer, and fall showed 0–20% variation, which is indicative of LST stability.

### Relationship between habitat suitability and seasonal temperature trends in habitats

**Fig 6:**
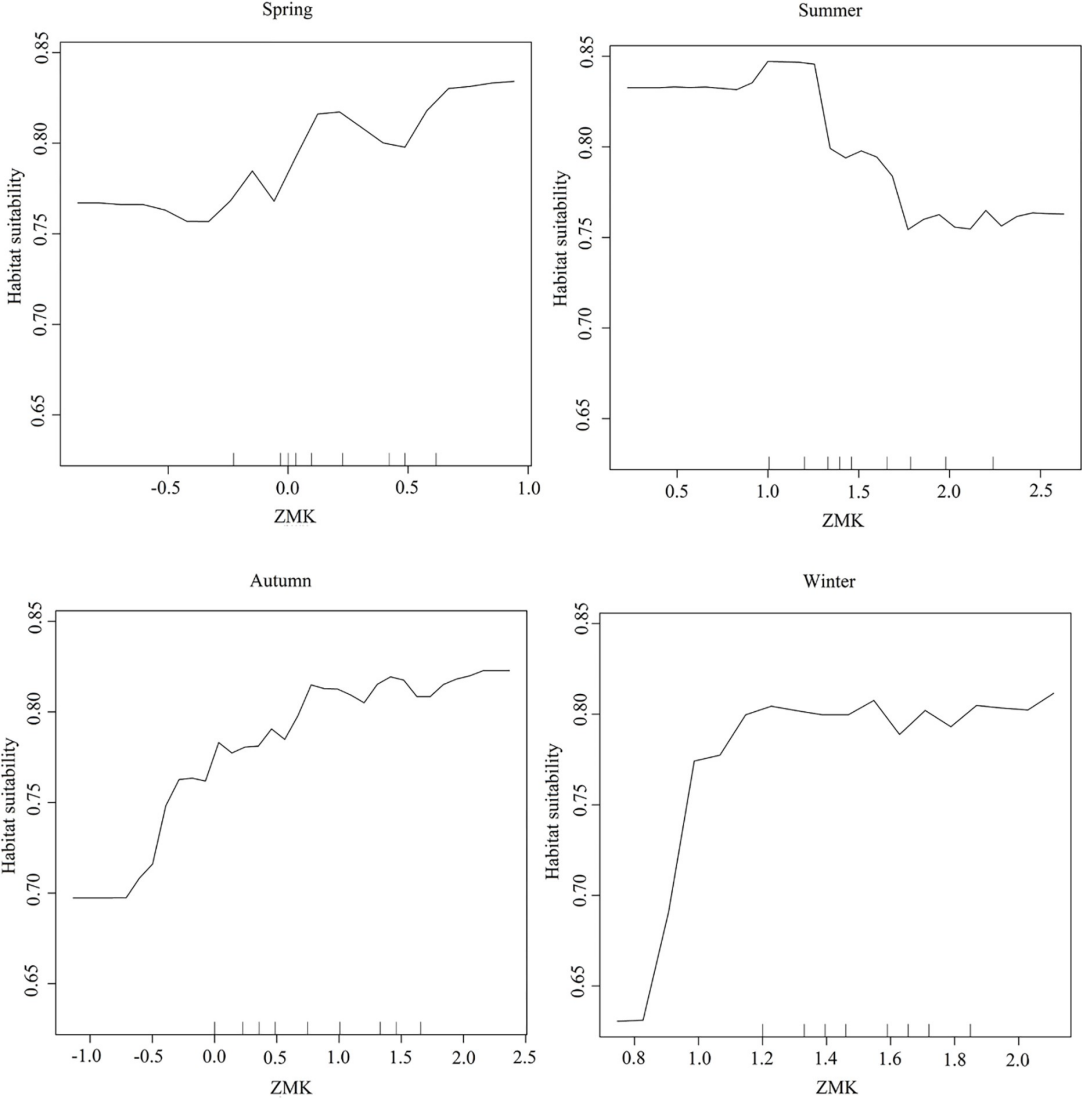
ZMK index fluctuations and habitat suitability of *M*. *peregrina* across seasons.

Performance evaluation for the RF model indicates that the model captured the relationship between the variables well. The model could effectively display the changes in habitat suitability along with fluctuations in ZMK across different seasons (Table 2). Fig 6 shows the response curves depicting the relationship between ZMK and habitat suitability. In these graphs, the x axis represents ZMK, and the y axis represents habitat suitability. It should be noted that in all seasons, an increase in ZMK enhances suitability, except in summer, when suitability decreases. For example, the model predicts an increase in habitat suitability due to increased ZMK in spring. However, due to the limited extent of ZMK changes (ZMK < 1.96), the increased temperature did not significantly affect habitat suitability. In contrast, during summer (ZMK > 1.96), changes in temperature significantly reduced habitat suitability to less than 0.75.

**Table 2:** RF model performance based on the out-of-bag (OBB) error statistic.

Name	OBB
**RMSE**	0.14
**MSE**	0.02
**MAD**	0.11
**MAPE**	0.17
**R-SQ**	0.01

## Discussion

Temperature is a crucial ecological factor that can significantly influence the ecological niche of species, as well seed germination, bud establishment, and biotic interactions [64]. Temperature is considered the primary element controlling plant phenology [65]. Changes in temperature can impact ecosystem processes by influencing canopy cover temperature, photosynthesis, water and nutrient absorption, and enzyme activity [66]. Consequently, many researchers recognize increased temperature as a trigger for habitat degradation, fragmentation, and destruction [54]. Temperature can also affect species abundance and activity patterns [67, 68]. In the current study, the seasonal cycle of temperature changes was investigated using LST data within the distribution range of *M*. *peregrina* in southern and southeastern Iran for the first time. Despite its value in habitat suitability research, remote sensing data is often neglected in ecological studies. However, the scalability of RS data allows for the investigation of a wide range of phenomena. The results of this study demonstrate that the temporal application of remote sensing data has remarkable adaptability to habitat suitability models and, as a result, the assessment of the extent of habitat damage.

This study aimed to investigate how seasonal temperature trends affect habitat suitability and how LST fluctuations influence habitats across landforms. According to TSS, the model was able to distinguish between presence and absence areas (Table 1). East-facing slopes and the coast of the Oman Sea were more suitable for the species’ establishment. Compared to arid areas, coastal areas experience less LST variation due to the monsoon weather, lower temperatures, and higher precipitation [69]. Consequently, these regions can provide more stable and conducive conditions for the establishment of *M*. *peregrina*. The results indicate that LST change trends vary across different seasons, showing increasing trends in spring, summer, fall, and winter. Generally, among the presence points, none of the regions exhibited an increasing trend in spring. Regions with LST changes follow diverse patterns; for instance, a number of small and scattered areas are located in the Makran coast, while a different group of areas tend to occur in the middle part and at high elevations during summer (Fig 3). This pattern could be related to the rapid temperature increase in drought-prone regions compare tod those closer to water bodies, leading to the distribution of regions with an increasing trend across all parts of the study area. Additionally, landform classes affected temperature fluctuations, with the most significant fluctuations belonging to mountain tops and upland drainages and the least fluctuations occurring in other landform classes.

Increased temperatures, shorter winters, and longer summers have been predicted for the northern hemisphere [70]. Temperature changes are the main determinant of leaf growth in early spring and the delayed loss of foliage in fall, although the impact on spring phenology is comparatively greater [71, 72]. For example, warmer winters lead to the acceleration of phenomena associated with spring, making it difficult for many species to meet their needs [73]. Moreover, increased winter temperatures may affect the time of flowering and the reproductive cycle. These conditions have been reported for alpine plants [74]. Moreover, warmer winters affect bud dormancy and consequently, plant growth [75, 76].

Within the scope of the current study, areas located in higher latitudes experienced an increasing LST trend due to lower elevation and humidity. Due to elevation, the seasonal humidity in the siutable habitat may vary. Aligned with this finding, reports indicate that humid seasons exhibit a stronger negative correlation with LST compared to dry seasons [77], likely due to higher humidity levels during those periods. In addition, an inverse correlation has been reported between LST and elevation in similar studies [78]. In contrast to spring and summer, the increasing LST change trend exhibited a scattered distribution pattern in fall and winter. Specifically, in winter, the increasing trend was observed towards the northeastern part of the species’ distribution range. On the other hand, there is a correlation between plant phenology (influenced by environmental factors) and LST, where phenological changes have cooling effects on LST during the growth period. Accordingly, an early start to the growth season and a delayed end of the growth season lead to surface cooling. These cooling effects are primarily caused by increased thermal transfer from the Earth to the atmosphere through reduced aerodynamic resistance [79]. Overall, it can be noted that spatial and temporal LST changes result from variations in the sun’s height and azimuth across different seasons [80]. In other words, as LST is a function of seasonal changes, it can be analyzed using trend analyses in the form of time series.

The study of the response curve from the RF regression model indicates that increasing trends in ZMK have a linear relationship with habitat suitability across all seasons (Fig 6). This suggests that suitable habitats for the species are under the influence of increasing LST trends. In spring, ZMK values are lower than the significance levels for z-scores. In fall, scattered changes are observed, and from summer to fall, changes are mostly observed in the northern and southern parts of the study area. During winter, vast areas of the study area are subjected to increasing LST changes, and compared to fall, changes become more dramatic, indicating that the northern and southern parts experience more substantial changes. From winter to spring, temperature changes are concentrated toward the south. Consequently, the seasonal cycle of temperature changes has affected the species’ distribution range in the study area. Investigating the impact of climate changes on the distribution of *M*. *peregrina* using climate variables indicated that the mean monthly temperature ranges had less fluctuation in climate change scenarios [46]. Thus, LST changes are different from predicting scenarios for climate changes.

Increased temperature is generally considered a favorable trend for *M*. *peregrina* as it can expand the distribution of the species [46]. Specifically, desert plants are capable of withstanding temperatures of up to 70°C [81]. Aligning with our findings, it has been reported that the species’ seeds germinate at up to 60°C [82]. Studying the impact of climate changes on this species demonstrated that variables such as the mean temperature of the warmest quarter and isothermality had the most significant impact on the species [83], indicating the importance of climatic factors such as temperature on the distribution of the species. Temperature and precipitation are two crucial factors that specify species distributions [84]; However, various combinations of these features in arid environments can pose significant challenges for plants and act as selective forces [85]. While the increased temperature can create suitable conditions, the negative impacts should also be considered. Increased temperature can reduce seed germination potential [86], which may occur through reduced water availability. In arid areas and deserts, water is a short-lived trigger for ecophysiological processes, including photosynthesis rate, dry matter production, carbohydrate allocation to roots, and primary and secondary growth [87]. Based on the results, the habitats of *M*. *peregrina* are exposed to temperature increases in fall and winter (Fig 6), leading to a potential reduction in precipitation. On the other hand, these changes in precipitation can affect the cambial activity of the species because of reduced water accessibility and temperature [88]; thus, changes in seasonal patterns may significantly influence cambial activity and wood formation [87].

Several studies have been conducted on the identification of microclimatic refuges within the species’ distribution range [48, 54]. However, these studies can be criticized from two aspects: 1. These studies use bioclimatic variables, which rely on interpolation and suffer from limitations in identifying microclimates and evaluating the relationship between habitats and environmental variables in mountainous areas. 2. The relationship between distribution range and potential refuge-worthy land cover elements (such as landforms) is not examined in these studies. As stated, available microclimate refuges are not fully captured by climate models used for vulnerability assessments [89, 90]. The results obtained from investigating the seasonal LST fluctuations in different landforms indicate that upland drainages experienced the least severe temperature fluctuations. These regions typically include water bodies and other sources of humidity [91], which reduce temperature fluctuations. Increased humidity can act as a buffer for LST, as shown by the inverse correlation between LST and surface humidity; specifically, the negative relationship grows more pronounced with increased surface humidity [80]. Moreover, rooting depth can increase in these regions. Similar to upland drainages, canyons have low temperature fluctuations and provide suitable environmental conditions for the species due to their complex structural features, high humidity, and reduced susceptibility to human activities [54]. For large-seeded species like *M*. *peregrina* with the highest post-dispersal, the set of mentioned conditions can be considered as a strategy against predation [92, 93]. Seed traits such as germination and weight are vital features of plants [94]. Seed germination is sensitive to environmental conditions and can only occur under suitable conditions [24], as the species exhibits flower abortion in very dry regions [95]. Thus, these landforms likely provide favorable conditions by influencing factors like humidity, temperature, and consequently, vegetation cover, which can itself reduce the heat stored in soil and surface structures through transpiration [96]. Aligning with this finding, it has been reported that *M*. *peregrina* is a glycophyte, establishing on lowland areas and slopes with a coarse soil texture and strong drainage [97].

Temperature fluctuations can also affect seed size and weight, as observed in *Potamogeton pectinatus* [98]. Arid plants are sensitive to parameters such as precipitation, topography, and landform [99]. In line with our findings, a study on habitat suitability fluctuations under various climate change scenarios has suggested that mountain tops and canyons will experience the largest fluctuations in LST [46]. This implies that regions with less temperature heterogeneity are more likely to undergo significant changes under the influence of climate change. Overall, the literature indicates the importance of landforms for the distribution of plant species [100]. Considering the evidence, it appears that the habitats of the species have been affected by seasonal temperature trends. Although extreme climatic conditions can determine the species’ distribution range [101], freezing tolerance will become less important as temperatures increase, and so the species may shift to higher elevations due to the warming trend. It seems that LST changes in the habitats have affected the species through parameters such as humidity, landforms, and phenology. Therefore, LST is proposed as a strong trigger influencing the species’ distribution range in the study area. Future studies should explore LST trends and the impact of LST on habitat quality and species’ distributions.

Proper selection of environmental variables and high spatial resolution are essential for ecological modeling [54]. When choosing a satellite for trend analysis, research objectives must be balanced against temporal fluctuations and spatial resolution. The spatial resolution should increase with the level of detail necessary for land cover analysis. Temporal fluctuations will also diminish with increasing spatial resolution. This study employed the MODIS/061/MYD11A1 product as a seasonal average since diurnal variations of LST are crucial in studies of climate change. One of the product’s shortcomings, the clear-sky bias, has been addressed by using mean LST values in the current study. If daily data from this product are to be used, this issue needs to be taken into account.

For instance, when a single image is required, it is advised to use the 8-day LST product. Low mobility and the small size of a species may determine the recommended spatial resolution for the study. Therefore, downscaling this data is possible if the use of average daily LST data is taken into consideration for seasonal studies of low-mobility species. When contrasted with the information generated from processing the model, the outcomes will be noteworthy. Apart from MODIS, LST can also be obtained from other satellites such as Sentinel and Landsat. Therefore, further data processing will be required in order to obtain greater details and lower temporal frequency.

## Conclusion

In this study, the impact of LST changes was investigated within the distribution range of *M*. *peregrina*. The analyses were conducted using non-parametric models, including the Mann-Kendall test, species distribution models, and the random forest algorithm to investigate LST dynamics. Seasonal changes have affected the entire range of the species, with the severity of changes being particularly pronounced in summer and winter. In all seasons, an increase in ZMK corresponded with enhanced habitat suitability, indicating increasing trends in the presence areas. The study also revealed the role of landforms in regulating temperature. Landforms such as local ridges and regions along the edge of the Oman Sea were suitable areas for potential range expansions due to the favorable humidity conditions.

More comprehensive studies should be conducted concerning LST trends in regions adjacent to the suitable habitats, taking into account landuse, the impact of human activities, and soil surface temperature. Moreover, it is proposed to develop a comprehensive program to avoid vegetation degradation and soil warming to protect this medicinal species. However, implementing such programs may face challenges due to environmental constraints and the social and economic conditions of the stakeholders. Thus, further research is recommended to clarify the impact of different climate scenarios while emphasizing the role of social and economic issues in the region and their effects on habitat suitability. Additionally, given the influence of climatic elements, soil conditions, and biological factors on growth and phenology [102, 103], phenological dynamics and triggers in Moringa species must be studied to improve phenological models and enhance our understanding of the carbon cycle in the ecosystem.

## Supporting information

S1 File https://figshare.com/articles/dataset/_b_Spatiotemporal_analysis_of_seasonal_trends_in_land_surface_temperature_within_the_distribution_range_of_b_b_i_Moringa_peregrina_i_b_b_Forssk_in_Southern_and_Southeastern_Iran_b_/26196041. (ZIP)

## Abbreviations

LST: Land surface temperature
SDM: Species distribution model
MODIS: Moderate Resolution Imaging Spectroradiometer
MK: Mann Kendall
CART: classification and regression tree
RF: random forest
GPS: generalized paths seeker
MARS: multiple additive regression
LR: logistic regression
DEM: Digital Elevation Model
PCA: principal component analysis
