# Supplementary figures and images for: Spatiotemporal analysis of seasonal trends in land surface temperature within the distribution range of Moringa peregrina (Forssk.) in Southern and Southeastern Iran
Source: PLoS One. 2024 Jul 25;19(7):e0306642. doi: 10.1371/journal.pone.0306642 (PMC11271878; doi:10.1371/journal.pone.0306642)

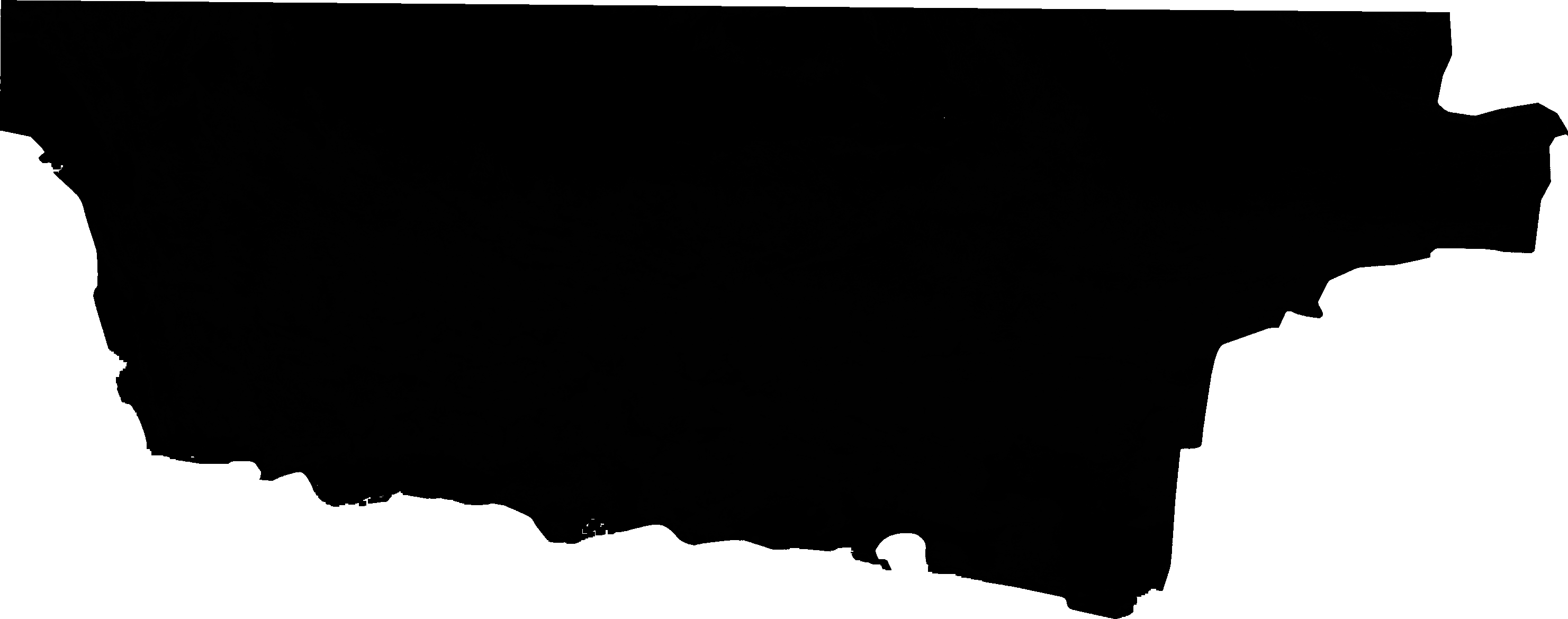

Supplement: S1 File — (ZIP) [file pone.0306642.s001.zip › S1/hsm.tif]

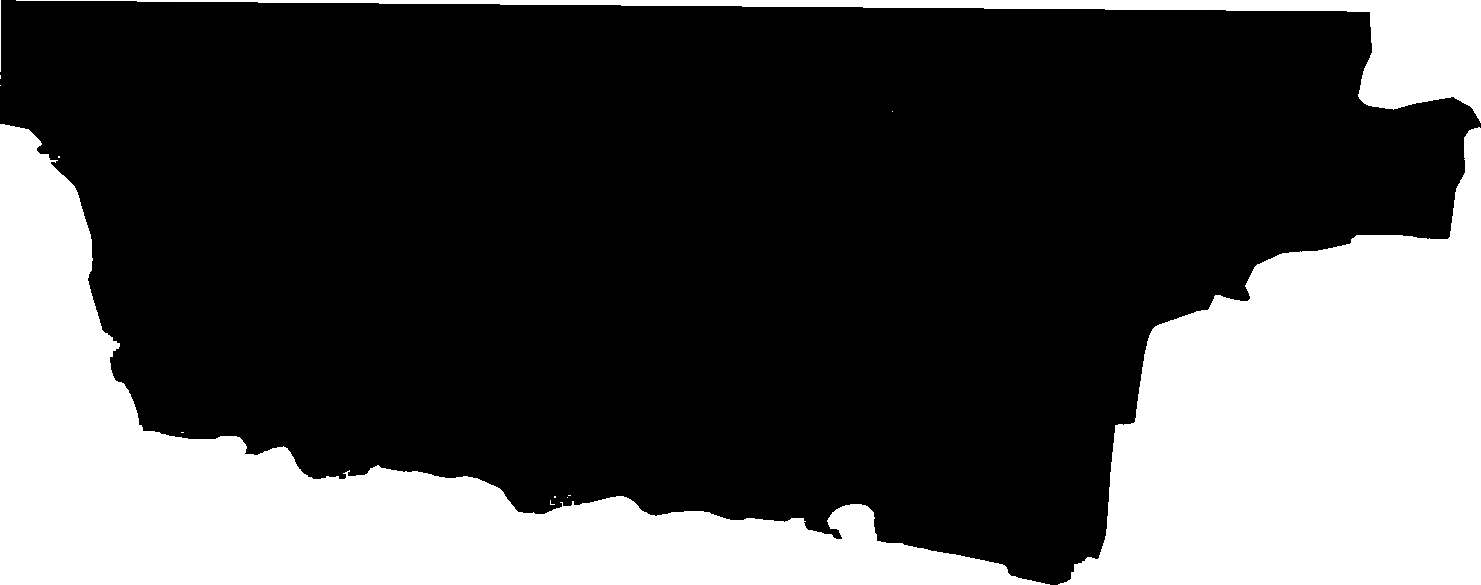

Supplement: S1 File — (ZIP) [file pone.0306642.s001.zip › S1/hsm.tif.ovr]
